# Androgen Receptor Point Mutations: A Mechanism of Therapeutic Resistance and a Framework for Rational Drug Design

**DOI:** 10.3390/cancers18061043

**Published:** 2026-03-23

**Authors:** Avan Colah, Sára Ferková, Han Zhang, Glenn Liu, Leonard MacGillivray, Pierre-Luc Boudreault, William Ricke

**Affiliations:** 1 Division of Pharmaceutical Sciences, School of Pharmacy, University of Wisconsin-Madison, Madison, WI 53705, USA; 2 Département de Pharmacologie-Physiologie, Faculté de Médecine et des Sciences de la Santé, Université de Sherbrooke, Sherbrooke, QC J1H 5N4, Canada; 3 Department of Medicine, School of Medicine and Public Health, University of Wisconsin-Madison, Madison, WI 53705, USA; 4 Département de Chime, Faculté des Sciences, Université de Sherbrooke, Sherbrooke, QC J1K 2R1, Canada; 5 Department of Urology, School of Medicine and Public Health, University of Wisconsin-Madison, Madison, WI 53705, USA

**Keywords:** therapeutic resistance, androgen receptor ligand-binding domain, point mutation, molecular mechanisms

## Abstract

**Background**: Point mutations to the androgen receptor (AR) ligand-binding domain (LBD) are becoming increasingly recognized as a mechanism of therapeutic resistance in castration resistant prostate cancer (CRPC). The present review explores how point mutations induce molecular changes that contribute to the eventual treatment failure of androgen receptor pathway inhibitors (ARPIs) in CRPC. **Methods**: The PubMed database was searched for structural studies on the AR LBD. Eligible articles included molecular docking analysis and emphasized changes in ligand–receptor interactions after point mutation. Structural data were obtained from the Protein Data Bank (PDB) using the search parameters “Androgen receptor ligand binding domain”, “Homo sapiens”, and “X-ray diffraction”. PDB files of wild-type and point mutant AR LBDs were accumulated for analysis. **Results**: A functional shift from inhibiting to activating AR has been documented for multiple ARPIs. Crystallography data and in silico evaluation have deciphered how changes in steric hindrance of the AF-2 domain contribute to ARPI loss of function. To combat therapeutic resistance, discovery efforts have begun to consider combination approaches of orthosteric and allosteric inhibitors, as well as compounds that target other AR domains. Although lead compounds have been identified, none have progressed into the clinic. **Conclusions**: Questions remain regarding the best approach for rationally designing new AR targeting therapeutics. Understanding how structural changes to the AR LBD lead to the failure of clinical therapeutics is a necessary step that should precede drug discovery campaigns. Moreover, computational modeling is a powerful tool that should be leveraged to streamline therapeutic development.

## Simple Summary

Point mutations in the androgen receptor ligand-binding domain are a clinical cause of drug resistance in castration resistant prostate cancer. This review examines how androgen receptor point mutations alter the structure of the ligand-binding domain and switch the function of some therapeutics from blocking receptor signaling to activating signaling. Structural studies demonstrate how point mutations change ligand–receptor interactions, with particular emphasis on changes that occur in the AF-2 region. Inability of ligands to inhibit the motility of the AF-2 region has been identified as the common cause of resistance across multiple clinical therapeutics and point mutations. To overcome resistance, researchers are exploring a combination of orthosteric and allosteric inhibitors and drugs targeting other receptor regions, although clinical translation has not been achieved. Critically, a deeper understanding of point mutations, supported by molecular docking and computational modeling, is essential for designing the next generation of androgen receptor targeting therapeutics.

## 1. Introduction

Prostate cancer (PCa) is the second most frequently diagnosed malignancy in men worldwide and is one of the principal contributors to the health burden in the aging male population [1]. The highest incidence rates of PCa are observed in men over 65 years of age, and PCa is projected to cause 11% of cancer-related deaths among US men in 2025 [2,3,4]. Targeting metastatic PCa with androgen deprivation therapy (ADT) is the cornerstone treatment. Despite improvements in diagnostic techniques and primary interventions, there is no curative measure for patients who progress to metastatic castration resistant prostate cancer (CRPC). At the molecular level, CRPC is primarily driven by dysregulation of androgen receptor (AR) signaling pathways, which includes mutational events [5]. Detailed reviews discussing these molecular mechanisms of AR-positive and AR-negative disease progression have been published throughout the CRPC literature [6]. Although administration of second-generation androgen receptor pathway inhibitors (ARPIs) like enzalutamide, apalutamide, and darolutamide, or CYP17 inhibitors including abiraterone acetate can prolong patient survival, the vast majority of patients eventually develop disease progression despite enhanced androgen receptor pathway inhibition [7,8,9].

One of the mechanisms by which AR-dependent CRPC has been reported to acquire therapeutic resistance is through point mutations to AR. Although over 1000 AR mutations have been identified, only a fraction have been shown to be clinically significant. Sequencing of advanced-stage prostate cancers revealed that 44% of CRPCs had genomic alterations to AR, with 20% involving point mutations [10]. Some of these point mutations have been reported to alter at or near the key F877 residue within the ligand-binding domain (LBD), which has roles in the H-bonding of both natural and synthetic AR agonists, including testosterone (T), dihydrotestosterone (DHT), and Metribolone (R1881) [11,12,13]. Point mutations that alter the AR LBD often emerge due to the selective pressure of therapeutic intervention with androgen receptor antagonists [14,15,16,17,18]. Structural rearrangements following point mutations enable larger ligands, including estradiol (E) and other steroid hormones, to gain access to the LBD and activate AR [14]. Critically, AR point mutation can also convert androgen receptor antagonists to behave like agonists, thereby negating their ability to inhibit AR signaling. Examples of this pharmacological switch have been reported post-treatment with both first- and second-generation ARPIs, including the flutamide-induced T878A mutation [14,15] and the bicalutamide-induced W742C mutation [16]. Although second-generation ARPIs were developed to maintain AR antagonism despite resistance mechanisms, long-term efficacy in this approach was not observed. This is exemplified by the F877L mutation induced by enzalutamide and apalutamide [17,18,19,20]. To improve disease outcomes, there is a critical need for drug discovery efforts to prioritize the development of a broad-spectrum AR antagonist with distinct properties which allow for long term efficacy.

In this review, we examine the implications of AR point mutations on ARPI functionality, with emphasis on computational analysis of molecular mechanisms. Additionally, we discuss the development of non-canonical AR inhibitors that target orthosteric or allosteric sites on the AR LBD. Literature on AR point mutations can differ in annotation based on the codon counts used. For example, Phenylalanine (F) can be located at either 876 (old method) or 877 (new method) codons, thus making F876L and F877L equivalent point mutations. The same can be applied to the L701H/L702H, W741L/W742L, T877A/T878A, and others. Throughout this review, our discussion maintained the codon counts reported in the primary research articles or Protein Data Bank (PDB) files used for analysis. Our summary aims to provide a comprehensive understanding of the mechanistic and therapeutic significance of AR point mutations in prostate cancer and to outline future directions for rational drug design.

## 2. Androgen Receptor Structure and Molecular Function

**Figure 1:**
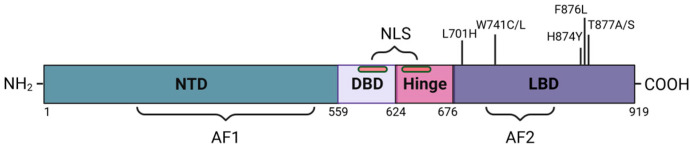
Schematic representation of androgen receptor structure, including major functional domains: N-terminal domain (NTD), DNA binding domain (DBD), the hinge region, and the ligand-binding domain (LBD). Key regions indicated through the length of the receptor include the activation function domains 1 (AF-1) and 2 (AF-2), along with the nuclear localization signal (NLS). Within the LBD, key point mutations are marked with black reference lines.

AR is a nuclear transcription factor and a member of the steroid hormone receptor superfamily [21]. Ligand-activated AR functions as a dimerized protein complex to activate transcription. In accordance with other nuclear receptors in its class, the three major functional domains of AR include: the N-terminal domain (NTD), the DNA binding domain (DBD), and the C-terminal ligand-binding domain (LBD) (Figure 1) [22]. The NTD accounts for the majority of AR transcriptional activity and houses the activation function-1 (AF-1) domain, which is constitutively active [23]. Within the inner region of AR, the DBD functions to facilitate the binding of AR dimers with promoter DNA and to induce transcriptional activity. Consistent with other nuclear receptors, the DBD consists of two zinc fingers that directly bind DNA [24]. A bipartite nuclear localization signal (NLS) has also been identified at the junction between the DBD and the flexible hinge region of AR. The NLS is responsible for the nuclear translocation of the dimerized receptor [25]. Lastly, the LBD is composed of 11 α-helices and the activation function 2 (AF-2) domain (a hydrophobic surface composed of helices 3, 4, and 12), which provides a unique conformation where activating ligands and their cofactors can bind to AR [22,23,26,27]. Helix 12 (H12) has emerged as a critical component of AF-2 and acts as a lid to stabilize ligands within the LBD [22,27]. Focusing on the LBD, X-ray crystallography data of ligand/AR LBD complexes determined that steroidal ligands (T, DHT, and R1881) interact with four key amino acids through hydrogen bonding: N705, Q711, R752, and T877 [11,12,13]. Elucidation of these structural characteristics has been essential for understanding the mechanisms of AR signaling and the distinct features which provide specificity to receptor activation and inhibition.

**Figure 2:**
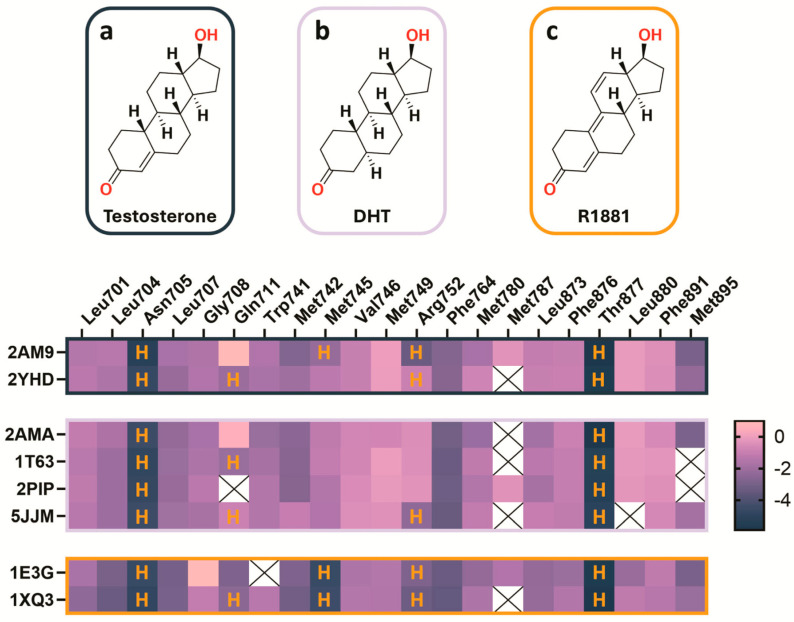
Interaction energy-based heatmap of T (**a**), DHT (**b**) and R1881 (**c**) within the AR LBD. Heteroatoms, along with their bound hydrogen atoms (e.g., hydroxyl groups), are annotated in red. Below are heatmaps with outlines color matched to their associated ligands T (black), DHT (light pink), and R1881 (orange). The vertical axis lists the corresponding PDB entries, while the upper horizontal axis denotes individual AR residues. Color intensity ranges from pink to dark blue, reflecting interaction energies in kcal/mol. Residues annotated with H in the heatmaps indicate hydrogen bond interactions contributing to ligand recognition. Structural data were obtained from the Protein Data Bank PDB IDs: 2AM9, 2YHD, 2AMA, 1T63, 2PIP, 5JJM, 1E3G, and 1XQ3.

The comparative interaction energy-based heatmap generated for the eight co-crystallized AR–ligand complexes, classified according to their PDB entries, confirms a high degree of consistency among structurally related steroidal ligands (Figure 2). Specifically, the ligands T, DHT, and R1881 all target the canonical binding site and interact with 19–21 binding-site residues, reflecting a binding mode largely driven by hydrophobic interactions. In addition, hydrogen bonding residues, including Asn705, Gln711, Met745, Arg752, and Thr877, exhibit highly conserved interaction patterns across these three steroidal ligands. Point mutations affecting this highly conserved binding mode cause significant alterations in ligand binding affinity and receptor activation.

## 3. Categories and Implications of AR LBD Point Mutations

Point mutations to the AR LBD can alter the susceptibility of AR to activation by endogenous ligands, including T and DHT. Work assaying transactivation of point-mutated AR across a range of DHT concentrations delineated five classes of functional impacts from point mutation [28]. These classifications describe point mutations found in high-grade PCa samples along the full length of the AR protein, where 62% (28 of 45 mutations) induce loss of function to DHT, and 16% (7 of 45 mutations) give rise to constitutive activity. Within the LBD alone, 61% (14 of 23 mutations) result in insensitivity to DHT and cluster between residues 720 and 798 [28]. Additional specifications of each of these mutations, which alter AR transactivation, have been extensively reported [28].

**Figure 3:**
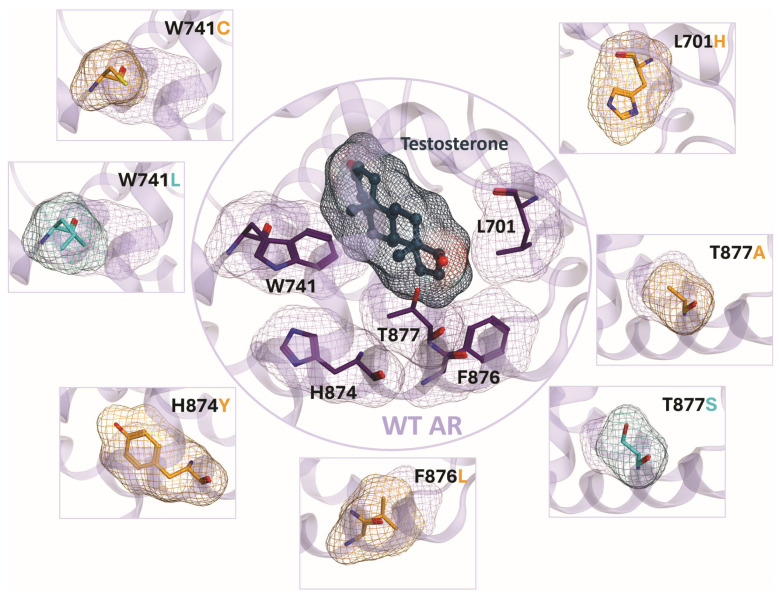
Clinically relevant point mutations within the ligand-binding domain (LBD) of the human AR. The central panel illustrates testosterone bound in the canonical ligand pocket, with wild-type (WT) residues L701, W741, H874, F876, and T877 shown in dark purple stick mode. The corresponding lipophilic surface is depicted as a purple mesh. Testosterone is displayed in green-blue stick mode, while the electronegative mesh denotes the ligand volume occupied in the pocket. Surrounding panels provide comparative views of the lipophilic surface for WT residues (purple mesh) and the respective point mutations, highlighted in orange or turquoise. The AR backbone shown in purple corresponds to the crystal structure PDB 2AM9.

In the context of prostate cancer, the discussions of AR point mutation often center around the conferral of therapeutic resistance. Here, we will focus on point mutations, which are the most clinically relevant due to emergence after therapeutic intervention with ARPIs. When evaluating the incidence of point mutations arising in PCa patients, several mutations were frequently and reproducibly detected. Separate investigations profiled circulating tumor DNA in 429 patients with metastatic CRPC, 892 patients with advanced stage PCa, and meta-analyses of 1614 CRPC patients [29,30,31]. Although the proportion of patients with specific point mutations varied based on sample size and heterogeneity, trends in frequency were consistent across studies. The L702H, T878A, and H875Y emerged as the most prevalent point mutations and were closely followed by W742C/L, T878S, and F877L [29,32] (Figure 3). Notably, these hot spot mutations to AR were common across all ethnicities profiled [32]. In the study employing a heterogeneous group of 892 advanced-stage PCa samples, it was determined that 18% of patients profiled had both point mutation and gene amplification to AR [29]. This combination exacerbates therapeutic resistance by increasing the abundance of point-mutated AR within CRPC cells. The detection of multiple co-occurring point mutations to AR has also been reported from this group of patients, including: L207H + H875H, T878S + T875Y, T878S + W742C, W742C + W742L, and L702H + T878A [29]. Individual contributions of co-occurring point mutations remain below 5% of total patients in the cohort. However, the prevalence of co-occurring point mutations is anticipated to increase as patients undergo successive rounds of therapeutic intervention. Each of these studies relied on measurements of ctDNA and it is critical to note that these assays do not have the ability to detect gene deletions as a category of mutation event [29]. This fact, in addition to limitations from study-to-study sample heterogeneity, indicates that results should not be interpreted as direct representations of real-world populations. Nevertheless, ctDNA profiling has provided clinically relevant insights into the mutational landscape of AR.

Quantification of AR point mutations in active patient populations is dependent on how clinicians implement diagnostic tests when acquired resistance emerges. Therefore, it is difficult to gauge the prevalence of AR point mutations in real-world populations. However, statistics from clinical trials focusing on metastatic CRPC (mCRPC) provide insights into current trends. Ongoing trials on ODM-208 (NCT03878823 and NCT03436485) were completed in cohorts where patients had demonstrated CRPC disease progression on one or more ARPIs [33,34,35]. Here, the most frequent AR point mutations identified were: L702H (15%), T878A (12%), H875Y (7%), V716M (2%), F877L (2%), and W742C (1%) and reported to be consistent with previous reports globally [34,35]. The anti-tumor activity at trial endpoints was especially observed in patients with AR mutations (73.7% response rate), which composed 42.6% of the overall study population [35]. Prospectively, ODM-208 has the potential to offer unique therapeutic utility in CRPC patients where AR point mutation has limited the types of drugs that will maintain efficacy in these subtypes of CRPC.

## 4. Molecular Mechanisms Underlying Therapeutic Resistance After Point Mutation

Crystal structures of human wild-type and point-mutated AR LBD have been utilized to improve our understanding of AR ligand functionality [13]. Computational profiling of the binding modes of ARPIs in complex with WT or point-mutated AR LBDs revealed that helix 12 (H12) is essential for antagonistic activity [36,37,38,39]. Molecular docking studies are available in the literature for flutamide, bicalutamide, apalutamide, and enzalutamide with respective point-mutated LBDs. The common theme attributed to the therapeutic failure of ARPIs and the transition of ARPIs from AR antagonists to AR agonists was the inability of ligands to sterically hinder H12 after a point mutation alters the structure of the LBD (Table 1).

Residues prone to mutation are typically involved in key binding interactions with AR ligands; these alterations can drive therapeutic resistance (Table 1). For example, hydroxyflutamide (HF)-associated resistance mutations can occur at L702 and T877. These residues are located at or in close proximity to L704, N705 and T877, which are necessary for HF to function as an AR antagonist [32,39]. Free energy decomposition analysis demonstrates that HF/T877A AR complexes induce a shift in HF binding compared to WT, where the energetic contributions of N705 and M859 are significantly increased to compensate for the reduced influence of A877 after mutation from T877 [39]. The HF/T877A AR ligand docking shift renders HF a functional agonist rather than an antagonist because H12 is no longer blocked from generating the coactivator binding site at AF-2. This finding was observed when HF was modeled in AR LBDs of T877A, F876L_T877A, and W741C_T877A subtypes [39]. Molecular docking studies of isolated and co-occurring point mutations of HF/W741C AR and HF/F876L AR did not convert HF from AR antagonist to AR agonist, demonstrating specificity of therapeutic resistance to HF [39]. Studies on the alignment of H12 in T877A AR LBD guided the development of the HF derivatives SC184 and SC333 [42] and drove the rapid generation of multiple compounds, which remain pure antagonists in both WT and T877A point-mutated receptors. This demonstrates how information from molecular docking studies can be implemented in rational drug design campaigns.

Bicalutamide (R-Bica), another first-generation ARPI, relies on binding to the W742 residue for function. Agonistic mutations W742L and W742C can arise in patients after long-term exposure to R-Bica [16,36]. In silico evaluation of W742C/L has been used to determine the molecular mechanism of R-Bica resistance [36,37]. For R-Bica/WTAR interactions, both the tryptophan residue and the sulfonyl linkage are large groups that destabilize interactions between M895 and R-Bica. As a result, the orientation of R-Bica sterically hinders H12 from closing, and an antagonistic LBD conformation is retained [36,37]. Upon substitution of the bulky tryptophan with small residues like leucine or cystine, R-Bica shifts closer to M895, and there is more room for H12 to close and form the AF-2 coactivator binding site. Therefore, the agonistic shift in R-Bica and reactivation of downstream AR signaling are attributable to increased interactions of R-Bica with the key residue M895 after point mutation [36,37]. This is further demonstrated in root-mean square fluctuation (RMSF) metrics, where the flexibility and mobility of H12 changed substantially when modeling R-Bica/W742C AR complexes compared to R-Bica/WT AR. When considering other AR point mutations—T877A, F876L, and F876L_T877A—R-Bica is not susceptible to these mechanisms of resistance [37].

**Figure 4:**
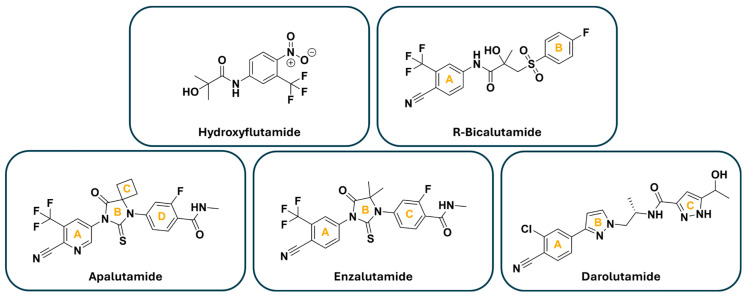
Chemical structures of select ARPIs with ring notation indicated in orange bold text.

Profiling enzalutamide after point mutation to the AR LBD revealed a similar trend in alterations to H12 flexibility. The F876L point mutation is characterized as conferring enzalutamide resistance and altering the AR LBD so that it is activated rather than inhibited upon enzalutamide binding. The F876 residue is known to be critical for enzalutamide binding to AR [38,43]. Molecular docking studies indicate that intermolecular hydrogen bonds between enzalutamide and AR are lost after the F876L and F876L_T877A mutation. Here, the interaction of enzalutamide with L876 is increased compared to that of F876 in WT AR [38]. The interaction brings the C-ring of enzalutamide (Figure 4) closer to H11, allowing H12 to close and form a coactivator binding site to facilitate transcription. Typically, the C-ring of enzalutamide is near H12 in WT AR and hinders H12 from closing, thereby blocking transcription [22,38].

In addition to the F876 residue, some reports profiling plasma samples from PCa patients suggest that H875Y, T878A, and T878S induce partial agonistic activity of enzalutamide—likely due to proximity to the F876 residue [44]. These mutations are also reflected in the clinic, where H875Y, F876L, and T877A have all been reported to induce the therapeutic failure of enzalutamide [40]. Specifically, H875Y and T877A are prone to induce a pharmacological switch when enzalutamide is dosed at high concentrations [45]. Molecular docking studies on enzalutamide/AR H875Y, /AR F876L, and /AR T877A complexes concluded that H11 and H12 were less flexible than WT, which resulted in enzalutamide being pushed out of the position it typically occupies [40]. These changes destabilize binding of enzalutamide and allow for robust coactivator binding after AF-2 formation and subsequent receptor activation [40]. Notably, this mechanism of action is not only consistent between H875Y, F876L, and T877A for enzalutamide, but it is also a theme across each of the therapeutics discussed.

## 5. Discovery Efforts for Alternative Orthosteric AR LBD Inhibitors

Prioritizing a mechanistic understanding of therapeutic resistance is critical for enabling the evolution of a new generation of therapeutics for CRPC. Computational approaches to molecular simulations have advanced substantially in recent years and have greatly assisted in deciphering molecular mechanisms. In addition, our summary of in silico modeling retrospectively highlights the utility of examining existing therapeutics to define a mechanistic rationale for resistance. However, computational tools (molecular simulations, docking, dynamics, mutational modeling) can also be applied to forecast the effectiveness of new drug candidates in disease models. One example profiles the effort to overcome the F876L point mutation, which can arise from enzalutamide or apalutamide exposure. Computational modeling was used to inform the systematic synthesis of a series of analogs with increasing complexity on the B-ring of the enzalutamide scaffold [41]. The new extensions from the B-ring were defined as D-ring substituents. The same strategy was previously reported to generate effective antagonists of WT AR [46]. Remarkably, the analog DR105 effectively inhibited AR F876L and WT AR. Moreover, transplanting effective D-rings from the enzalutamide scaffold to the apalutamide scaffold also resulted in AR F876L inhibition [41]. This is likely due to the overlap of the identical A-rings shared between the two molecules. Although the approach provides a model for rapid and targeted drug design, it is unlikely to solve the core issue underlying the relationship between ARPIs and point mutation emergence. Therefore, it is necessary to incorporate approaches apart from canonical small molecules that interact with the AR LBD.

Candidate compounds targeting AR LBD point mutations are not limited to those that are rationally designed from existing drug scaffolds. For example, a series of AR antagonists with a tetra-aryl cyclobutane core structure was derived and tested in CRPC models with point-mutated AR [47]. The cyclobutane series has a remarkably distinct structure from traditional ARPIs, which translates to a unique mechanism of action [47]. Despite promising preclinical data, these compounds have yet to progress to clinical trials. Here, in silico modeling may best be applied to gain molecular insights into the functionality of experimental therapeutics. Moreover, profiling of ligand-receptor complexes could be applied to determine intermolecular interactions that are aligned with traditional ARPIs compared to those that allow for the distinct functionality of the CB class of AR antagonists.

## 6. Coadministration of Orthosteric and Allosteric AR LBD Inhibitors

As previously described, first- and second-generation ARPIs interact with the AR LBD to inhibit AR signaling. Functionally, this is an indirect mechanism of inhibition where blockade of the AF-2 domain formation is a secondary consequence of ARPIs binding to the AR LBD. As ARPI-associated resistance mutations continue to emerge under selective pressure, the next question is whether directly targeting AF-2 could provide a viable therapeutic alternative. Although not at the forefront of current drug discovery efforts, the A2-F binding site remains a promising target to prevent further point mutations, which confer therapeutic resistance [40]. To date, drug discovery campaigns and virtual chemical screens have begun to elucidate chemical structures with AF-2 antagonistic capacity [48,49]. The AF-2 is a protein–protein interaction site, although challenging to target, that offers an attractive option for direct inhibition of AR transcriptional coactivation. Application of high-throughput screening identified six AF-2-selective compounds which exhibit IC_50_ values in the 4–36 µM range as measured by eGFP AR transcriptional assay [48]. Analysis of the docking poses of these six compounds highlighted key molecular interactions with residues Lys720, Glu893, and Glu897 within the “charged-clamp” region of the AF-2 site [48]. Ligand docking of AF-2 binders to the “charged-clamp” region directly blocks the anchoring of AR coactivators [48]. Identification of ligand binding potential at these key residues serves as a guide for further drug discovery and optimization.

**Figure 5:**
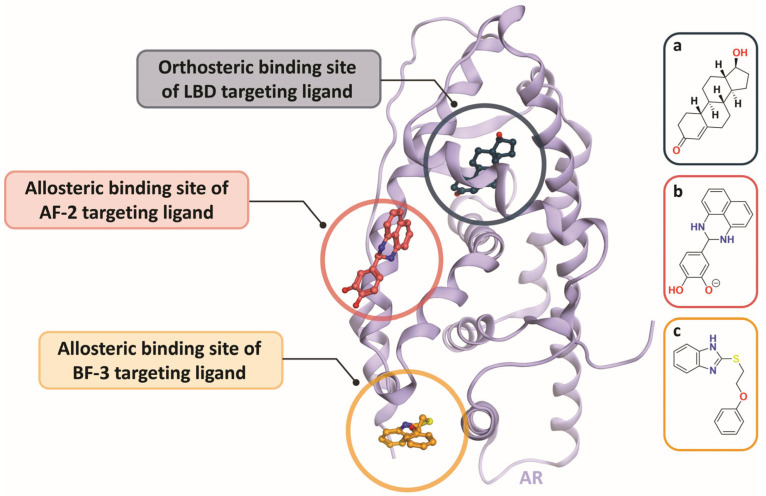
Ribbon representation of the human AR LBD highlighting orthosteric and allosteric ligand binding sites. Canonical AR agonists and antagonists, including testosterone (**a**), bind the orthosteric site (green-blue). The AF-2 allosteric site (red), targeted by the small-molecule inhibitor 4-(2,3-dihydro-1H-perimidin-2-yl)benzene-1,2-diol (**b**), was identified from the crystal structure PDB 2YHD (44). The BF-3 allosteric site (orange), targeted by 2-[(2-phenoxyethyl)sulfanyl]-1H-benzimidazole (**c**), was resolved from PDB 4HLW (46). The AR structure shown in purple corresponds to the crystal structure PDB 4HLW.

A secondary AR allosteric site, which has been implicated as a target for rational drug design, is the binding function-3 (BF-3) [50,51]. However, like AF-2, BF-3 has proven challenging to target due to the large but shallow size and high flexibility of the region [52]. The potent, selective, and metabolically stable BF-3 inhibitor VPC-13789 achieves favorable molecular interactions with the BF-3 domain through favorable H-bonding interactions between the amide-NH and the carbonyl of Asn833 within the AR LBD [51]. Based on this data, further optimization of biopharmaceutical potential led to the development of the prodrug VPC-13822, which exhibits enhanced bioavailability in murine models [51]. A comparative analysis of candidate AR inhibitors suggested that the BF-3 is a more compelling target compared to AF-2, owing to its higher sequence conservation across ARs [51]. Studies on each of these allosteric AR inhibitors provide foundational efforts to establish alternative mechanisms for long-lasting AR inhibition (Figure 5).

Computational studies of allosteric AR inhibitors have revealed that targeting a single site with multiple drugs can slow the emergence of resistance [48,53]. Extending this concept beyond AR, the combined use of allosteric and orthosteric agents has shown promise to overcome therapeutic resistance [54]. In the context of the androgen receptor, this translates to a combination approach with ARSIs as the orthosteric inhibitors and AF2 or BF3 as the allosteric compounds. Computational assessment of this approach was completed to determine its relevance in CRPC. Modeling of pharmacological combinations included DHT and bicalutamide at the orthosteric site paired with either candidate AF2 or BF3 inhibitors [54]. Notably, when modeled in combination with orthosteric ligands, the binding capacity of BF3 ligands was preserved while the AF2 allosteric site was perturbed when the orthosteric site was occupied [54]. Although CRPC remains a challenging disease to therapeutically target outside of the LBD, these studies offer promising insight into the potential of alternative pharmacological interventions.

## 7. Alternative Approaches for Targeting AR

In addition to the LBD, other regions of AR have emerged as therapeutic targets to disrupt AR signaling. While a comprehensive summary of AR-targeted therapeutic agents has already been published [55], our goal is to provide brief insights into alternative approaches that have gained traction in preclinical development. Many of these therapeutics were initially developed to target ARV7, which lacks the LBD entirely. However, these drug candidates also have broader relevance to AR point mutants. Critically, targeting alternative domains of AR has the potential to alleviate the selective pressure that drives point mutation emergence within the LBD.

Elimination of AR protein as a strategy for inhibiting AR-driven disease progression has been approached at the gene and protein levels. Application of antisense oligonucleotides or siRNA to target AR mRNA and reduce protein translation has shown promise in preclinical studies [56,57,58]. At the protein level, proteolysis-targeting chimeras (PROTACs) have been designed to target the DBD and NTD of AR. PROTACs are a type of bifunctional small-molecule compound that recruits E3 ubiquitin ligases to the protein of interest, thereby promoting its proteasomal degradation. Select examples include A031 [59], A9 [60], Au-AR pep-PROTAC [61], ARCC-4/ARV-110 [62,63], ARD series [64,65,66], MTX-23 [67], TD-802 [68], SNIPER-51 [69], and UT-34 [70]. PROTAC design takes advantage of the ubiquitin-protease system to strategically eliminate disease-driving proteins from target tissues. In the context of AR therapeutic resistance, PROTACs offer viable routes for eliminating the selective pressure to AR, which typically drives the emergence of resistance point mutation.

The PROTACs ARV-110 and ARV766, aimed at degrading AR in CRPC, have progressed into phase II clinical trials. ARV-110 was granted Fast Track designation by the FDA in 2019 and became the first PROTAC AR degrader to complete phase I/II trials in patients with mCRPC in 2025 (NCT03888612) [63]. However, results from this trial have yet to be posted. Separate investigations measuring the efficacy of orally administered ARV766 (Novartis) either alone or with abiraterone to mCRPC patients are currently active (NCT05067140). These trials highlight advancements of PROTACs in clinical stages of CRPC therapeutic development.

The AR DBD contains two α-helices: the P-box, or “recognition helix,” which directly binds to the transcription factor motifs of target genes, and the D-box, which mediates AR dimerization [71,72]. Small molecules that disrupt AR–DNA interactions by targeting the P-box, such as 4-(4-phenylthiazol-2-yl)morpholine and its analogs, have been shown to repress the transcriptional activity of AR [73]. Importantly, although DBD is structurally conserved among other nuclear receptors, including the progesterone receptor (PR), glucocorticoid receptor (GR), and estrogen receptor (ER), certain 4-(4-Phenylthiazol-2-yl) morpholine and its analogs still displayed specificity for AR [73]. Moreover, the D-box has been identified as a viable target for small-molecule inhibitors such as VPC-17160 and VPC-17281, which interfere with AR dimerization [74].

The AR NTD also serves as a crucial structural region that mediates AR dimerization, DNA binding, and transcriptional regulation [75,76,77]. To date, several small molecules targeting AR NTD have been developed and validated in vitro, including EPI-001/-002/-7170, ASR-600, biaryl isoxazole compound 16, and SC428 [78,79,80,81]. These small molecules exhibited the ability to bind to the NTD of AR.

The NTD AR inhibitor Masofaniten (EPI-7386) has progressed into clinical trials for hormone-sensitive and CRPC. In phase I/II trials evaluating coadministration of Masofaniten with Enzalutamide, treatment efficacy and acceptable safety profiles were observed in patients with treatment-naïve metastatic PCa at the primary endpoint of this study (NCT06312670) [82]. Although CRPC remains a challenging disease to therapeutically target outside of the LBD, these trials offer promising insight into the clinical translatability of these alternative pharmacological interventions.

Targeting AR outside of the LBD has grown in interest over the past few decades, with the goal of reducing the selective pressure on the AR LBD, which is responsible for the vast majority of resistance mechanisms. Since AR point mutations occur less frequently in the AR DBD and NTD than in the LBD, targeting these domains represents a promising strategy to overcome point mutation-mediated drug resistance in CRPC. Nonetheless, further preclinical and clinical evaluation is required to determine the efficacy, specificity, and safety of these emerging compounds.

## 8. Conclusions and Perspectives

The traditional small-molecule drug discovery process, constrained by chemical complexity, extensive timelines, and high costs, is undergoing profound reconfiguration, with emphasis on high-throughput screening methodologies to accelerate early-stage identification of therapeutic candidates. [83] Particularly in the field of AR-targeted early-stage drug discovery, computer-aided drug design (CADD) has become an integral component, incorporating X-ray structure-based analyses, molecular docking, or molecular dynamics simulations [84]. The breadth of approaches to CADD allows it to be implemented across many stages of the drug development pipeline. Using structure-based methods (molecular docking, homology modeling, fragment-based screening, and De novo drug modeling design) allows researchers to focus on methods where the 3D structure of the drug target is known. For example, molecular docking is most useful for optimization of small molecules since it provides a route for simulating interactions between small molecules and target proteins at the atomic level. Techniques like fragment-based screening are useful for strategically developing drugs that successfully target “undruggable targets”. Here, small fragments which initially bind weakly to a given target are identified [85]. These fragments are then merged using linkers to yield potent, novel drug modalities [85]. Notably, the development of PROTAC follows this model [86]. On the opposite end of the spectrum, quantitative structure-activity relationships (Q-SAR) aim to predict the function of molecules based on known structural features [87]. Consistent with traditional SAR, there is a correlation between structural features and known physiological implications [87]. Therefore, rational drug design can be centered around enhancing the functionality of lead molecules in development. As CADD technology develops, so does the potential for applying drug discovery efforts to non-canonical ligand binding sites. This aptitude is highly advantageous for the identification and feasibility profiling of new target domains, including in AR-targeted therapeutics.

In the context of CRPC, the vast majority of AR-targeting therapeutics bind to the LBD with a highly similar binding mode, resulting in a shared mechanism of inhibition. As a result, each generation of ARPIs offers advances in potency, but is prone to treatment failure with the emergence of a point mutation. Molecular docking studies elucidated that ARPI binding to the LBD actually serves as an indirect mechanism of inhibiting AF-2; it was quickly hypothesized that AF-2 could be directly targeted [40]. Consistent with our prior discussion, the rapid discovery of AF-2 and subsequently BF-3 ligands provides a prime example of how novel compounds can be strategically developed through structure-based lead optimization [51]. Critically, these two allosteric sites were not identified as viable pharmacophores prior to molecular docking analysis. These studies provide two examples of how incorporating structural biology and computational methods into rational drug design not only enhances ligand discovery but also expands our knowledge of pharmacologically relevant target sites.

In CRPC research and beyond, we are currently experiencing a transformative shift in approaches to rational drug design. Artificial intelligence (AI) and big data tools for drug discovery have begun to emerge as an impactful approach capable of reducing numerous barriers within the pharmaceutical development pipeline [88]. The theoretically accessible chemical space of molecules, often estimated to contain around 1 × 10^60^ small-molecule candidates, hinders the efficiency of research efforts [89]. As traditional methods of drug discovery rely on iterative processes, the success rate for identifying therapeutics within the vast chemical space is low, and target identification is time-consuming and labor-intensive. Therefore, we and others anticipate that leveraging advanced algorithms integrated with established technology for CADD will allow for rapid virtual screening of drug candidates and will impose a paradigm shift to current drug discovery methodology [88,90]. However, it is critical to note that many limitations to AI-mediated drug discovery have been proposed, including: (1) limited access to abundant high-quality structural data to inform AI-driven platforms, (2) technical challenges to algorithms, and (3) the lack of standardized objectives to apply as benchmarks for molecular success [88]. Despite these complications, the robust success of systems developed for AI-driven modeling of biomolecular interactions—like AlphaFold3—exhibits remarkable future potential [91]. For optimal success in drug discovery, researchers should not consider AI platforms as standalone tools. Rather, they should be used to accelerate discovery efforts without sacrificing the rigorous standards currently held within the pharmaceutical pipeline. Therefore, a strategic balance must be established between components of human-driven and AI-guided research. We anticipate that computational methods will continue to complement drug discovery research and ultimately stand to provide substantial benefit to clinical advancement and patient outcomes across many disciplines, including CRPC.

## Figures and Tables

**Table 1:** Summary of results from in silico modeling elucidating the mechanistic rationale for therapeutic failure of ARPIs.

Therapeutic Compound	Point Mutation	Alteration to Ligand Binding	Mechanism of Resistance	Source Files
Flutamide	T877A	The hydroxyl group of flutamide interacts with H11 instead of H12	Increased proximity of H12 to the AR LBD generates a coactivator binding site at AF2	PDB ID: 2AXA, 2AX6 [39]
Bicalutamide	W741L	Orientation of M895 becomes more stable and the B-ring of R-bicalutamide shifts toward L741 and away from H12	H12 is stabilized and able to move closer to the LBD and form an agonistic conformation	PDB ID: 2AXA, 2AX8, 1Z95 [36]
Bicalutamide	W741C	Steric clash between the B-ring of R-bicalutamide and H12 is reduced	Closed conformation of H12 generating a coactivator binding site at AF2	PDB ID: 2AXA, 1Z95 [37]
Enzalutamide	F876L	The C-ring of enzalutamide is oriented close to H11 instead of H12 after loss of pi stacking and van der Waals contacts.	Reorganization of H12 and formation of a coactivator binding site at AF2	PDB ID: 2AXA, 1Z95, 2Q7L [38,40,41]
Enzalutamide	H875Y—high concentrations	Compact ligand binding pocket pushes out enzalutamide and allows for inward movement of H12	H12 is in a tighter conformation, allowing for robust coactivator binding	PDB ID: 2AXA [40]
Enzalutamide	T877A—high concentrations	Compact ligand binding pocket pushes out enzalutamide and allows for inward movement of H12	H12 is in a tighter conformation, allowing for robust coactivator binding	PDB ID: 2AXA [40]
Apalutamide	F876L	Loss of favorable pi stacking and van der Waals contacts between the C-ring of apalutamide and F876 after mutation	Repositioning of H12 in conformation compatible with coactivator recruitment	PDB ID: 1Z95, 2AXA [41]

## Data Availability

We declare that no new primary research data was generated for this manuscript.

## Abbreviations

The following abbreviations are used in this manuscript:

ADT	Androgen Deprivation Therapy
AF-2	Activation Function 2
AR	Androgen Receptor
ARPIs	Androgen Receptor Pathway Inhibitors
BF-3	Binding Function 3
ctDNA	Circulating tumor DNA
CADD	Computer-Aided Drug Design
CRPC	Castration Resistant Prostate Cancer
DBD	DNA Binding Domain
DHT	Dihydrotestosterone
Enza	Enzalutamide
ER	Estrogen Receptor
eGFP	Enhanced Green Fluorescent Protein
GR	Glucocorticoid Receptor
H12	Helix 12
HF	Hydroxyflutamide
LBD	Ligand Binding Domain
NLS	Nuclear Localization Signal
NTD	N-terminal Domain
PDB	Protein Data Bank
PR	Progesterone Receptor
PROTACs	Proteolysis-Targeting Chimeras
R1881	Metribolone
R-Bica	R-Bicalutamide
RMSF	Root-Mean Square Fluctuation
T	Testosterone
WT	Wild Type
